# Supplementary material for: Regulation of angiogenesis by endocytic trafficking mediated by cytoplasmic dynein 1 light intermediate chain 1
Source: bioRxiv. 2024 Apr 1:2024.04.01.587559. Preprint. [Version 2] doi: 10.1101/2024.04.01.587559 (PMC11188074; doi:10.1101/2024.04.01.587559)
Supplement: Supplement 1 [file NIHPP2024.04.01.587559v2-supplement-1.pdf]

bioRxiv preprint doi: https://doi.org/10.1101/2024.04.01.587559; this version posted April 1, 2024. The copyright holder for this preprint (which was not certified by peer review) is the author/funder. This article is a US Government work. It is not subject to copyright under 17 USC 105 and is also made available for use under a CC0 license.

Johnson et al. Supplemental Figure 1

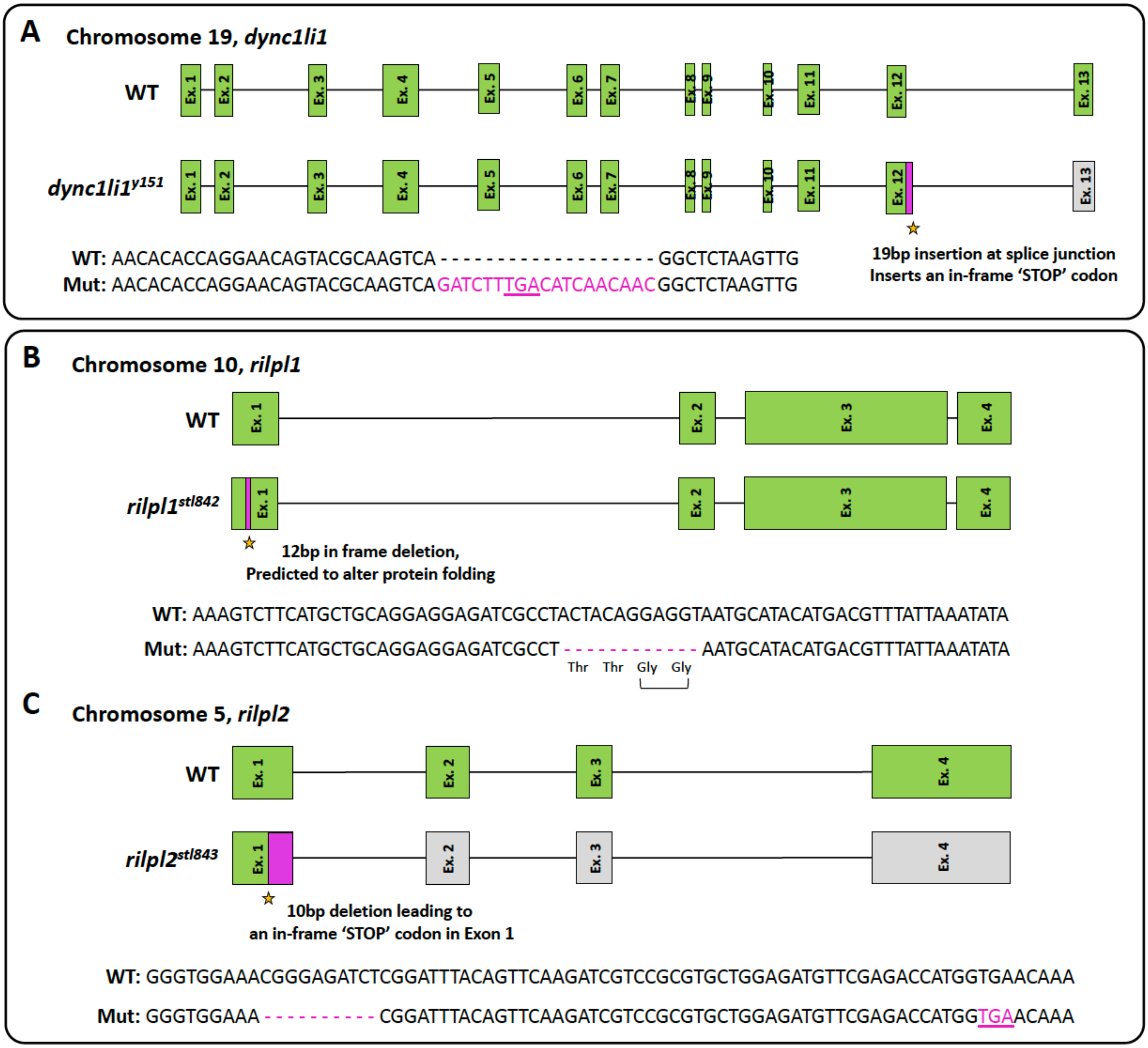

Supplemental Figure 1: Confirmation and identity of the *dync1li1*^{y151} and *rilpl1*^{stl842}/*rilpl2*^{stl843} mutations.
A. Schematic representation and sequence of the zebrafish *dync1li1* gene**.** The *dync1li1*^{y151} allele contains a 19 bp insertion at the end of exon 12 that introduces a premature stop codon, resulting in the elimination of exon 13. **B,C.** Schematic representation and sequence of the zebrafish *rilpl1*^{stl842} (D) and *rilpl2*^{stl843} (E) alleles. The *rilpl1*^{stl842} allele contains an in-frame 12 bp deletion in exon 1 that removes two glycines and is predicted to alter protein folding. The *rilpl2*^{stl843} allele contains a 10 bp deletion in exon 1 that introduces a premature stop codon.

bioRxiv preprint doi: https://doi.org/10.1101/2024.04.01.587559; this version posted April 1, 2024. The copyright holder for this preprint (which was not certified by peer review) is the author/funder. This article is a US Government work. It is not subject to copyright under 17 USC 105 and is also made available for use under a CC0 license.

Johnson et al. Supplemental Figure 2

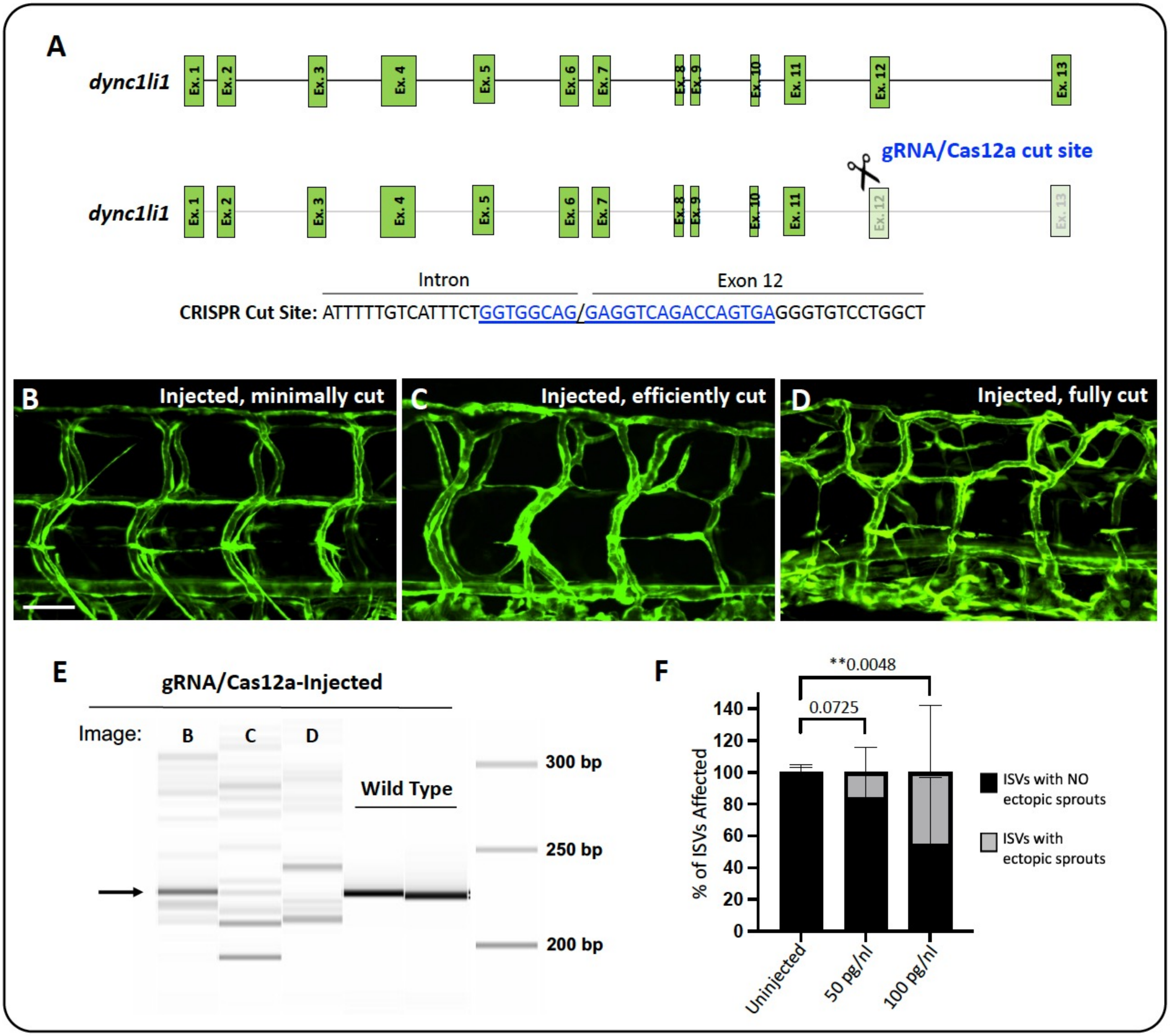

Supplemental Figure 2: *dync1li1* Crispants recapitulate the *dync1li1^{y151}* mutant phenotype.
A. Schematic representation of the zebrafish *dync1li1* gene**.** Cas12a and a gRNA targeted to the *dync1li1* exon 12 splice acceptor site was injected into the zebrafish at the 1 cell stage for analysis of phenotypes. **B-D.** Confocal images of ISVs in *dync1li1* Crispant zebrafish at 96 hpf. *Tg(fli:eGFP)* was used to label the vasculature. A gradation of ectopic ISV sprouting phenotypes are noted depending on the CRISPR cutting/editing efficiency per embryo. (B) Low cutting efficiency, mostly wild type presenting; (C) Efficiently cut but still some WT DNA left, induced ectopic sprouting; (D) Full cutting efficiency with no WT DNA present, induced a greater extent of ectopic sprouting. **E.** Fragment analysis of the individual gRNA/Cas12a-injected indicates the efficiency of indel generation in the genomic DNA. **F.** Percentage of ISVs displaying ectopic sprouts in uninjected controls and embryos injected with 50 or 100 pg/nL *dync1li1* gRNA. n=6-10 embryos. Statistics for panel F were calculated using a Kruskal-Wallis test (omnibus p-value= **0.0069) with Dunn's multiple comparisons test. Data are presented as the mean ± S.D. Images that are shown in B-D are from the 100 pg/nL injection dose. Scale bars: 50 um.

bioRxiv preprint doi: https://doi.org/10.1101/2024.04.01.587559; this version posted April 1, 2024. The copyright holder for this preprint (which was not certified by peer review) is the author/funder. This article is a US Government work. It is not subject to copyright under 17 USC 105 and is also made available for use under a CC0 license.

Johnson et al. Supplemental Figure 3

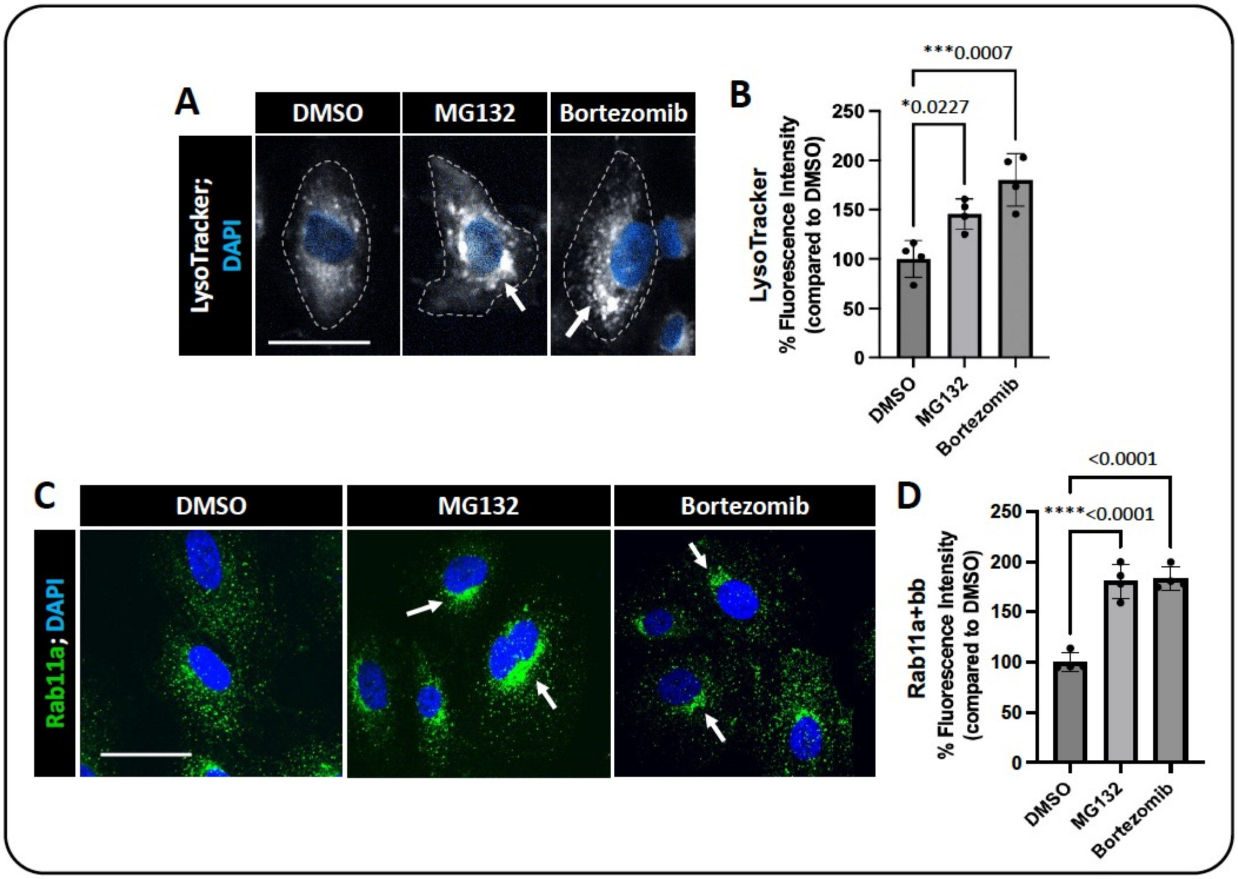

Supplemental Figure 3. Lysosome/proteosome inhibition increases levels of recycling endosomes and lysosomes within the perinuclear cloud.
A. Representative images of live HUVECs incubated with LysoTracker Deep Red for 1 hour after lysosome/proteosome inhibition. Cells treated with lysosomal/proteosome inhibitors show accumulation of the LysoTracker in the perinuclear cloud (arrows), suggesting disruption in lysosomal-mediated vesicle degradation and localization. Dashed lines highlight individual cell borders. **B.** Quantification of percent fluorescence intensity of LysoTracker in MG132 and Bortezomib treated HUVECs compared to DMSO control treated HUVECs. **C.** Representative images of HUVECs immunostained for Rab11a (green) following lysosomal-proteosome inhibition. Nuclei are shown in blue. HUVECs treated with inhibitors show an increase in Rab11a+ recycling endosomes in the perinuclear cloud (arrows). **D.** Quantification of percent fluorescence intensity of Rab11a positive vesicle in MG132 and Bortezomib treated cells compared to DMSO control treated cells. Statistics for panel B and D were calculated using a one-way ANOVA (omnibus p-values: B=****<0.0001, D=**0.0013) with Dunnett's multiple comparisons test. Data are presented as the mean ± S.D; n=4 experimental replicates. Scale bars: 50um
